# Retraction Note to: Association of metabolic syndrome with TyG index and TyG-related parameters in an urban Chinese population: a 15-year prospective study

**DOI:** 10.1186/s13098-022-00889-8

**Published:** 2022-08-19

**Authors:** Xin Zhang, Ting Zhang, Sen He, Shanshan Jia, Zhipeng Zhang, Runyu Ye, Xiangyu Yang, Xiaoping Chen

**Affiliations:** Department of Cardiology, West China Hospital, Sichuan University, No. 37 Guo Xue Alley, Chengdu, 610041 China

## Retraction Note: Diabetology & Metabolic Syndrome (2022) 14:84 https://doi.org/10.1186/s13098-022-00855-4

The Editor in Chief has retracted this article because it contains material that substantially overlaps with the following article [1].

Xiaoping Chen, Xin Zhang and Sen He do not agree to this retraction. Ting Zhang, Shanshan Jia, Zhipeng Zhang, Runyu Ye, Xiangyu Yang have not responded to any correspondence from the editor/publisher about this retraction.
